# Multiple Genome Constellations of Similar and Distinct Influenza A Viruses Co-Circulate in Pigs During Epidemic Events

**DOI:** 10.1038/s41598-017-11272-3

**Published:** 2017-09-19

**Authors:** Andres Diaz, Douglas Marthaler, Cesar Corzo, Claudia Muñoz-Zanzi, Srinand Sreevatsan, Marie Culhane, Montserrat Torremorell

**Affiliations:** 1 College of Veterinary Medicine, University of Minnesota, Saint Paul, 55108 United States of America; 2 School of Public Health, University of Minnesota, Minneapolis, 55454 United States of America

**Keywords:** Influenza virus, Viral epidemiology, Viral evolution, Viral transmission

## Abstract

Swine play a key role in the ecology and transmission of influenza A viruses (IAVs) between species. However, the epidemiology and diversity of swine IAVs is not completely understood. In this cohort study, we sampled on a weekly basis 132 3-week old pigs for 15 weeks. We found two overlapping epidemic events of infection in which most pigs (98.4%) tested PCR positive for IAVs. The prevalence rate of infection ranged between 0 and 86% per week and the incidence density ranged between 0 and 71 cases per 100 pigs-week. Three distinct influenza viral groups (VGs) replicating as a “swarm” of viruses were identified (swine H1-gamma, H1-beta, and H3-cluster-IV IAVs) and co-circulated at different proportions over time suggesting differential allele fitness. Furthermore, using deep genome sequencing 13 distinct viral genome constellations were differentiated. Moreover, 78% of the pigs had recurrent infections with IAVs closely related to each other or IAVs clearly distinct. Our results demonstrated the molecular complexity of swine IAVs during natural infection of pigs in which novel strains of IAVs with zoonotic and pandemic potential can emerge. These are key findings to design better health interventions to reduce the transmission of swine IAVs and minimize the public health risk.

## Introduction

Influenza A viruses (IAVs) can infect many animal species including humans and pigs, and are considered a major public health risk because zoonotic infections can cause human pandemics^1^. Swine are considered a dominant species for global IAV delivery^2^ and pig movements within and between countries contribute to the increased diversity of swine IAVs^3–5^. However, the epidemiology, diversity and molecular evolution of IAVs during natural infection of pigs are not clearly understood. Understanding the mechanisms that allow IAVs to evolve and persist in swine populations is crucial to design health interventions that minimize viral transmission between pigs and from pigs to people.

IAVs are Orthomyxoviruses that contain eight single stranded negative sense RNA gene segments namely polymerase B2 (PB2, segment 1), polymerase B1 (PB1, segment 2), polymerase A (PA, segment 3), hemagglutinin (HA, segment 4), nucleoprotein (NP, segment 5), neuraminidase (NA, segment 6), matrix (M, segment 7), and non-structural protein (NS, segment 8). The main antigenic proteins (HA and NA) are used in a dual classification system to differentiate viruses into seventeen HAs (H1-H17) and nine NAs (N1-N9)^5–7^. Aquatic birds are considered the natural reservoir for most IAVs and at least 116 HA-NA IAV combinations have been isolated from avian species^1^. However, only few IAV subtypes are endemic in humans and pigs.

A reassortant IAV caused the 2009 IAV pandemic and highlighted the risk of swine IAVs for public health^8^. In North America, the diversity of swine IAVs has increased due to multiple reassortment events and the introduction of human IAVs including the 2009 pandemic virus^9–11^. During infection of pigs or humans the IAV genome replicates as a dynamic “swarm” of genotypes closely related to each other^12,13^. Moreover, different IAV subtypes can coexist in pig populations, and the same IAV subtype can persist for prolonged periods of time^14–16^. In the USA H1N1, H1N2, and H3N2 IAVs are endemic in pigs and cluster in six H1 clades (α, β, γ1, γ2, δ1, and δ2) and four H3 clusters (I, II, III and IV)^10,17,18^. Nevertheless, the diversity and evolution of IAVs at the herd level in the contemporary swine industry are not clearly understood.

According to the United States Department of Agriculture (USDA), National Agricultural Statistical Service (NASS), in the USA there are almost 72 million pigs and approximately 9 to 10 million are slaughtered every month^19^, which illustrates the high turnover rate of swine populations. The majority of these pigs are weaned at 3 weeks of age from farrow-to-wean herds and moved to growing pig production sites (i.e wean to finish farms) where they are reared until market (~24 weeks of age). Additionally, pig farms can be managed as continuous or all-in/all-out flows. In a continuous flow, pig batches are moved in and out of production sites on a continuous basis with pigs present on-site all the time. In contrast, in all-in/all-out flows, a batch of pigs is used to fill the production site and no more pigs are added until all pigs from the previous batch are removed. Since production flows and sites might influence the evolution of swine IAVs it is crucial to study the dynamics of the virus at the herd level.

We hypothesize that the persistence of swine IAVs in pigs after weaning is associated to the plasticity of IAV genome and the continuous influx of infected and susceptible individuals. Therefore, we designed a prospective cohort study and used next generation sequencing (NGS) technologies to characterize the complete genome of the IAV population during natural infection of pigs after weaning. We found that three different influenza viral groups (VG) co-circulated at different proportions over time and produced two overlapping epidemic events in which the majority of the pigs had recurrent infections ﻿with﻿ IAV. These results are important because they unraveled a deeper layer of the epidemiology and molecular diversity of swine IAVs at the herd level and will help to design better health interventions to reduce the transmission of IAVs between pigs and from pigs to humans.

## Results

### Epidemiological findings

One hundred and thirty-two 3-week old pigs were randomly selected and identified from a group of weaned pigs (N = 2200) at arrival (week 0 (W0)) to a commercial wean-to-finish-farm. Pigs were weaned from a single IAV positive farrow-to-wean farm, kept together with the remaining pigs in the batch, and sampled weekly for 15 weeks (W1 to W15). Five pigs (3.4%) died at weeks 3, 7, 8, 11 and 14 and their cause of death was not determined. A total of 2,080 individual nasal swabs were collected over 15 weeks, and 369 (17.7%) tested positive for IAV by real time reverse transcriptase polymerase chain reaction (RRT-PCR). At weaning (W0), 27 pigs (20.5%) tested IAV positive by RRT- PCR and the 15-week period prevalence of IAVs infection was 98.4% (n = 130). Only two pigs (1.6%) tested IAV negative throughout the study period, although one of them died in W3.

**Figure 1:**
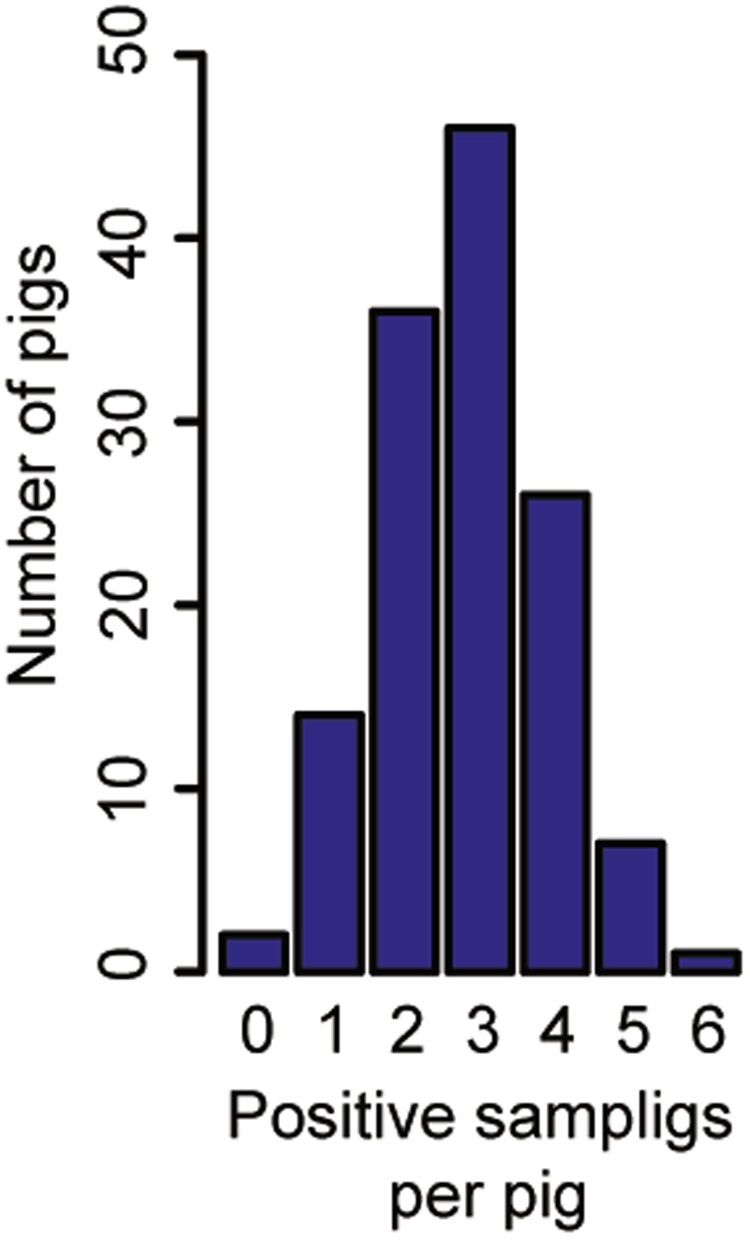
Number of pigs and sampling events where pigs tested positive to influenza A virus (IAV) by real time reverse transcriptase polymerase chain reaction (RRT-PCR). Each bar represents the number of pigs with 0 to 6 IAV positive swabs during the study period.

**Figure 2:**
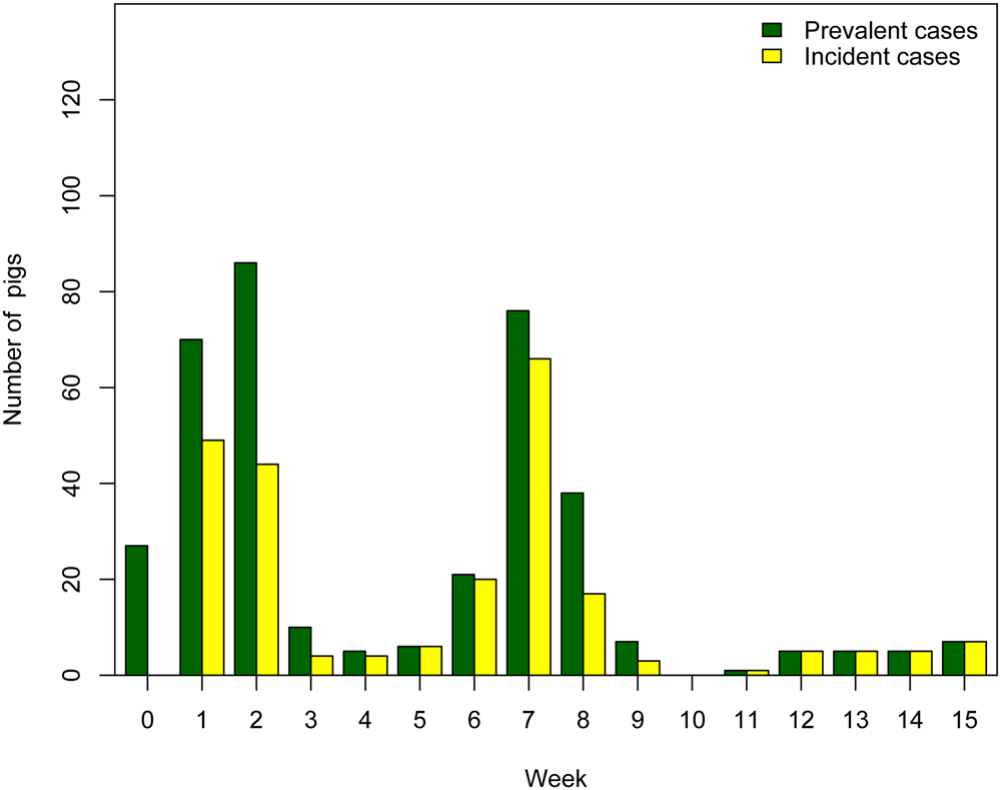
Prevalent and incident cases of influenza A virus distributed by week. Green bars represent the total number of real time reverse transcriptase polymerase chain reaction (RRT-PCR) positive cases per week (prevalent cases) and yellow bars illustrate the number of new cases found every week (incident cases).

One hundred and sixteen pigs (87.9%) tested positive to IAV more than once (Fig. 1) and 103 pigs (78%) had recurrent infections based on the study definition for recurrence (testing IAV positive in two non-consecutive weeks). There were two overlapping epidemic events of IAV infection during the study period (Fig. 2) with no statistical difference between the number of prevalent cases at the epidemic peaks in W2 and W7 (p = 0.24). During the 15-week period, the weekly prevalence of IAV infection ranged between 0% and 65.2% and the incidence density of IAV infection of pigs after weaning ranged between 0 and 71 cases per 100 pig-week (Table 1).

**Table 1:** Epidemiological findings. Number of pigs that tested influenza A virus (IAV) positive by real time reverse transcriptase polymerase chain reaction (RRT-PCR) distributed by week (W).

W	Total number of pigs	Prevalent cases (Prevalent rate per 100)	Incident cases (incident density per 100 pigs-week)
0	132	27 (20.5)	NA
1	132	70 (53.0)	49 (46.7)
2	131	86 (65.2)	44 (71.0)
3	131	10 (7.6)	4 (8.7)
4	131	5 (3.8)	4 (3.3)
5	131	6 (4.6)	6 (4.8)
6	130	21 (16.0)	20 (16.0)
7	130	76 (58.0)	66 (60.0)
8	129	38 (29.2)	17 (30.9)
9	129	7 (5.4)	3 (3.3)
10	129	0 (0)	0 (0)
11	128	1 (0.8)	1 (0.8)
12	128	5 (3.9)	5 (3.9)
13	128	5 (3.9)	5 (4.1)
14	127	5 (3.9)	5 (4.1)
15	127	5 (5.5)	7 (5.7)

### Identification of influenza viral groups

Ninety-two out of 369 positive swabs (25%) were selected for IAV genome amplification and deep genome sequencing using Illumina MiSeq as the next-generation sequencing (NGS) platform. After the first template-assembly, three different influenza A virus groups (VG) were identified (VG1, VG2, and VG3). At the HA level VG1, VG2, and VG3 clustered within swine H1-gamma, H1-beta and H3-cluster-IV IAVs respectively. At the NA level, VG1 and VG2 were N1 viruses while VG3 were N2 viruses. The pairwise percent identity (ClustalW) between the consensus sequences of all gene segments of three representative samples (n = 24) of these three VGs is shown in Table 2. These 24 templates were used to re-assemble all samples sequenced.

**Table 2:** Pairwise sequence comparison among influenza A virus (IAV) gene templates. Percent identity (ClustalW alignment) between gene segments of three representative IAVs from virus group 1 (VG1, H1 gamma), 2 (VG2, H1 beta), and 3 (VG3, H3 cluster IV) that were used to map all Illumina sequencing reads.

Segment	VG1 vs. VG2	VG1 vs. VG3	VG2 vs. VG3
1 (PB2)	95.4	94.9	95.7
2 (PB1)	95.4	95.7	95.6
3 (PA)	94.8	91.8	95.5
4 (HA)	90.7	51.8	52.4
5 (NP)	94.3	93.1	96.8
6 (NA)	95.5	53.3	51.8
7 (M)	88.5	88.1	98.0
8 (NS)	95.5	95.6	96.6

**Figure 3:**
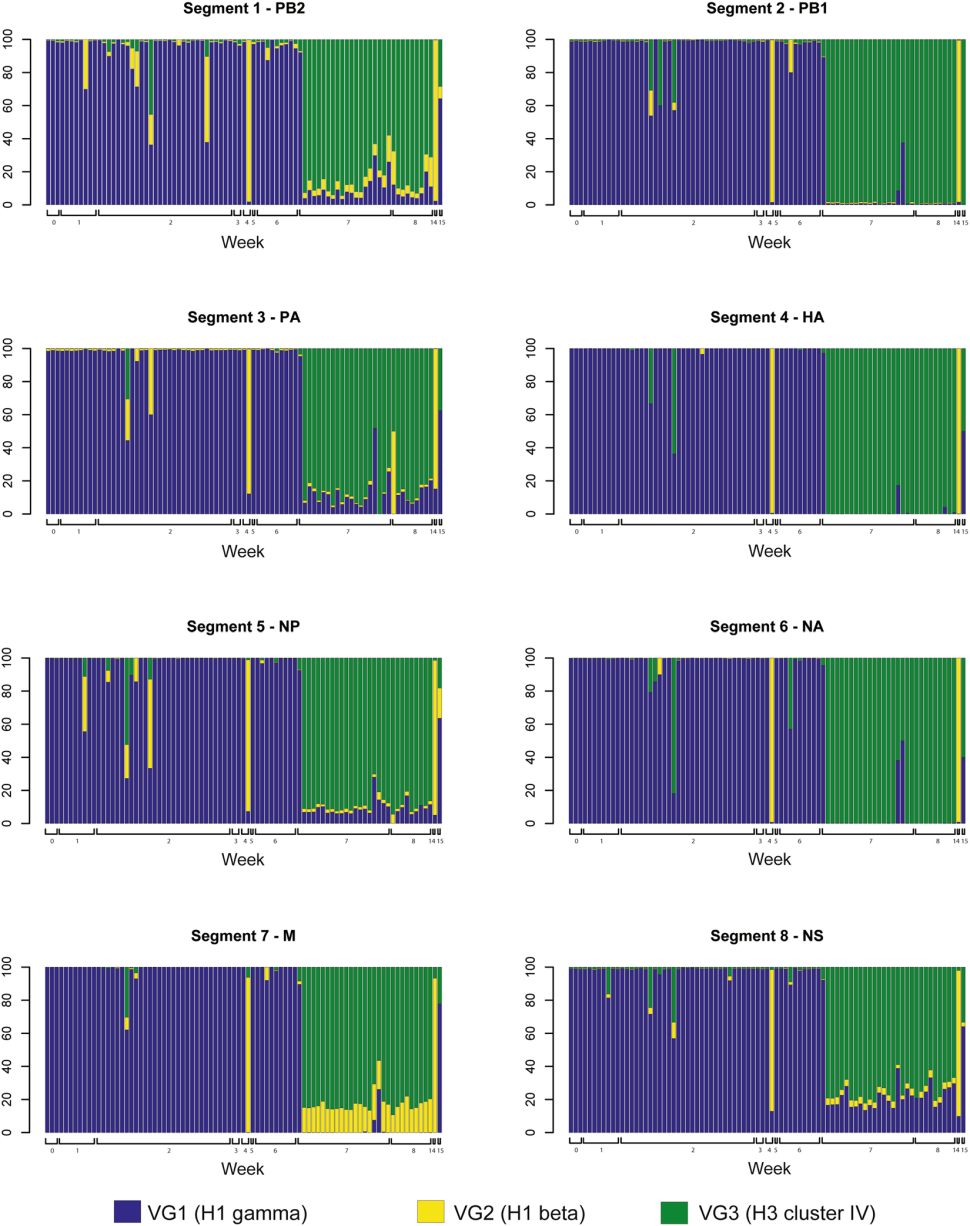
Percent distribution of Illumina sequencing reads for each influenza A virus (IAV) gene segment over time. Each bar represents a sample. Bars are color coded based on the percentage of Illumina sequencing reads (y-axis) mapped to IAV gene segments templates (1 to 8) of viral groups one (VG1), two (VG2) and three (VG3), and are distributed by week (x-axis).

After quality control, nine samples (10.8%) yielded only partial IAV gene contigs and were excluded from the analysis. From the remaining samples (n = 83), 13,559,009 Illumina sequencing reads were successfully mapped to the reference templates (Fig. 3). Overall, the majority of Illumina reads obtained from the first epidemic event of IAV infection mapped to the reference templates of VG1 (H1 gamma virus) while the majority of reads obtained after week 6 mapped to the reference templates of VG3 (H3 cluster IV virus). However, in most weeks we detected reads that mapped to at least two different VGs. Illumina reads mapping to the reference templates of VG2 only predominated in samples sequenced in W4 and W14 (Fig. 3). Overall, the sequencing assembling process yielded 649 complete IAV genes that were classified as VG1, VG2 or VG3 based on the sequence template mapped (Table 3), and had a mean coverage per position that ranged between 184X and 14,746X (Table 4) (GenBank accession numbers MF919672 - MF920318. There were no complete IAV gene segments obtained from W9 to W13 and W15.

**Table 3:** Frequency distribution of complete influenza A virus gene sequences by virus group (VG1, H1 gamma; VG2, H1 beta; and VG3, H3 cluster-IV) and gene segment. Polymerase base 2 (PB2), polymerase B1 (PB1), polymerase A (PA), hemagglutinin (HA), neuraminidase (NA), matrix (M), and non-structural protein (NS).

Virus	Gene segments								Total
	PB2 (%)	PB1 (%)	PA (%)	HA (%)	NP (%)	NA (%)	M (%)	NS (%)	
VG1 - H1 gamma	46 (60.5)	43 (59.7)	48 (64.0)	48 (61.5)	49 (62.0)	50 (60.2)	59 (63.4)	58 (62.4)	402 (61.9)
VG2 - H1 beta	2 (2.6)	2 (2.8)	2 (2.7)	2 (2.6)	2 (2.5)	2 (2.4)	4 (4.3)	4 (4.3)	19 (2.9)
VG3 - H3 cluster IV	28 (36.8)	27 37.5)	25 (33.3)	28 (35.9)	28 (35.4)	31 (37.3)	30 (32.3)	31 (33.3)	228 (35.1)
Total (%)	76 (100)	72 (100)	75 (100)	78 (100)	79 (100)	83 (100)	93 (100)	93 (100)	649 (100)

**Table 4:** Mean coverage per nucleotide position of IAV gene segments assembled. Numbers within brackets indicate the minimum and maximum coverage per position used to estimate the mean.

Segment	VG 1	VG 2	VG 3
1 (PB2)	1670 (10–10097)	1240 (67–1464)	1147 (2–10272)
2 (PB1)	850 (4–11570)	184 (41–494)	966 (2–5892)
3 (PA)	1919 (34–7475)	326 (4–584)	749 (6–442)
4 (HA)	2273 (29–7820)	1114 (218–2712)	1397 (5–4508)
5 (NP)	2797 (9–9732)	5264 (42–8212)	2610 (2–7639)
6 (NA)	3076 (16–10403)	3990 (1105–5999)	2735 (33–7193)
7 (M)	7946 (19–27708)	14746 (3692–24216)	9115 (4–25519)
8 (NS)	8804 (3–28868)	14338 (109–23785)	9697 (8–28848)

### Diversity of influenza A virus genome

**Figure 4:**
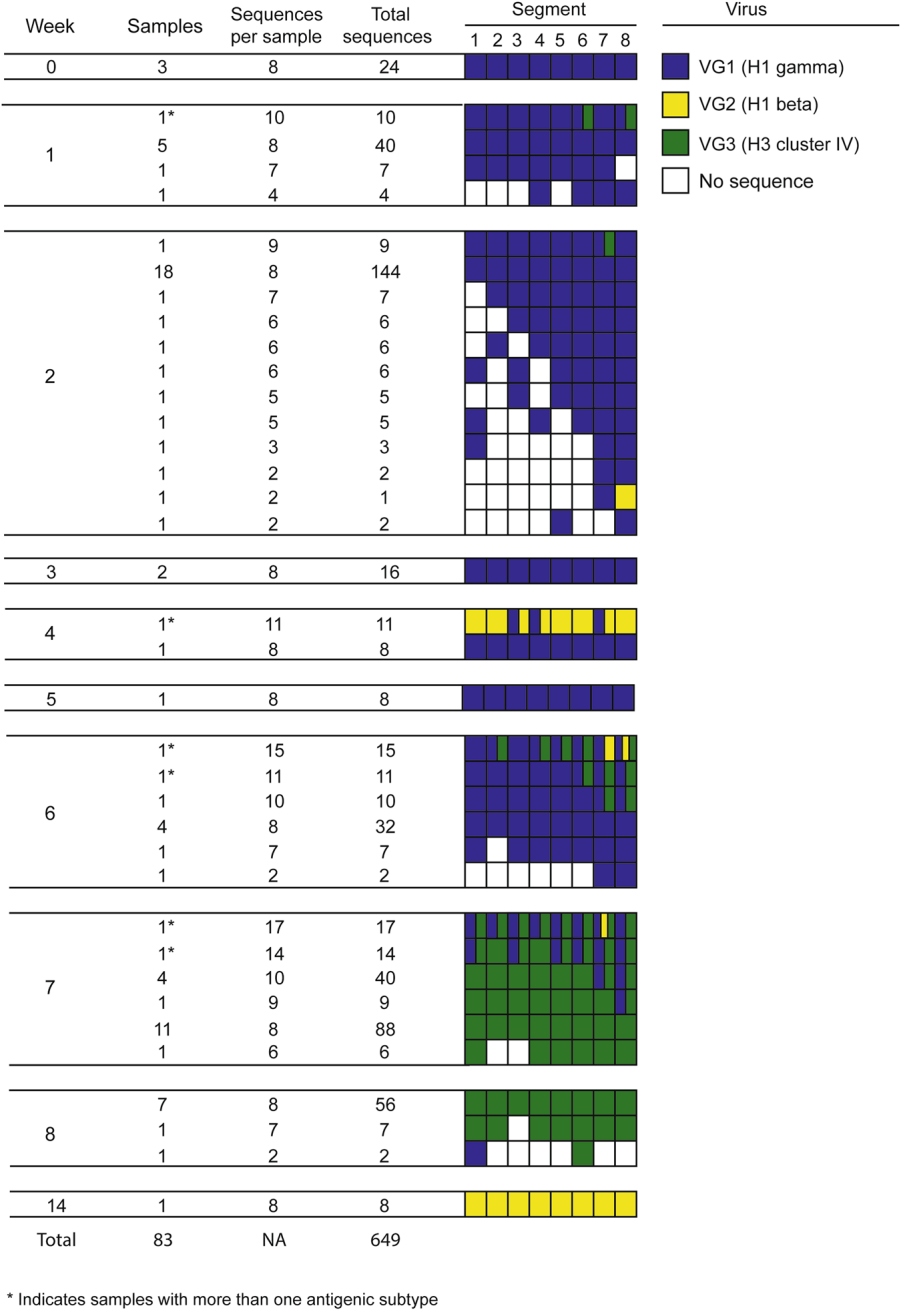
Influenza A virus (IAV) gene constellations distributed by week and sample. The first four columns indicate the week (W0 to W14), number of samples for each genome constellation found, number of complete IAV gene segment recovered per sample, and total number of complete gene sequences obtained. The remaining columns illustrate IAV gene segments assembled based on viral group one (VG1, blue), two (VG2, yellow), and three (VG3, green). White boxes indicate that it was not possible to assemble a complete IAV gene segment.

Thirteen distinct IAV genome constellations were recovered and demonstrated co-circulation (at the population level) and co-infection (at the individual level) of three clearly distinct VGs over time (Fig. 4). Complete IAV gene segments from a single VG were recovered at W0, W3 and W5, from two different VGs at W1, W4 and W8, and from all three VGs at W2, W6 and W7. Additionally, 68 samples (81.9%) contained complete gene segments from a single VG and 15 (18.1%) contained complete gene segments from more than one VG (VG1 and VG2, n = 2; VG1 and VG3, n = 11; and VG1, VG2, and VG3, n = 2). Furthermore, six samples contained more than one antigenic subtype (H1 gamma and H1 beta, n = 1; H1 gamma and H3 cluster IV, n = 2; and N1 and N2, n = 5).

**Figure 5:**
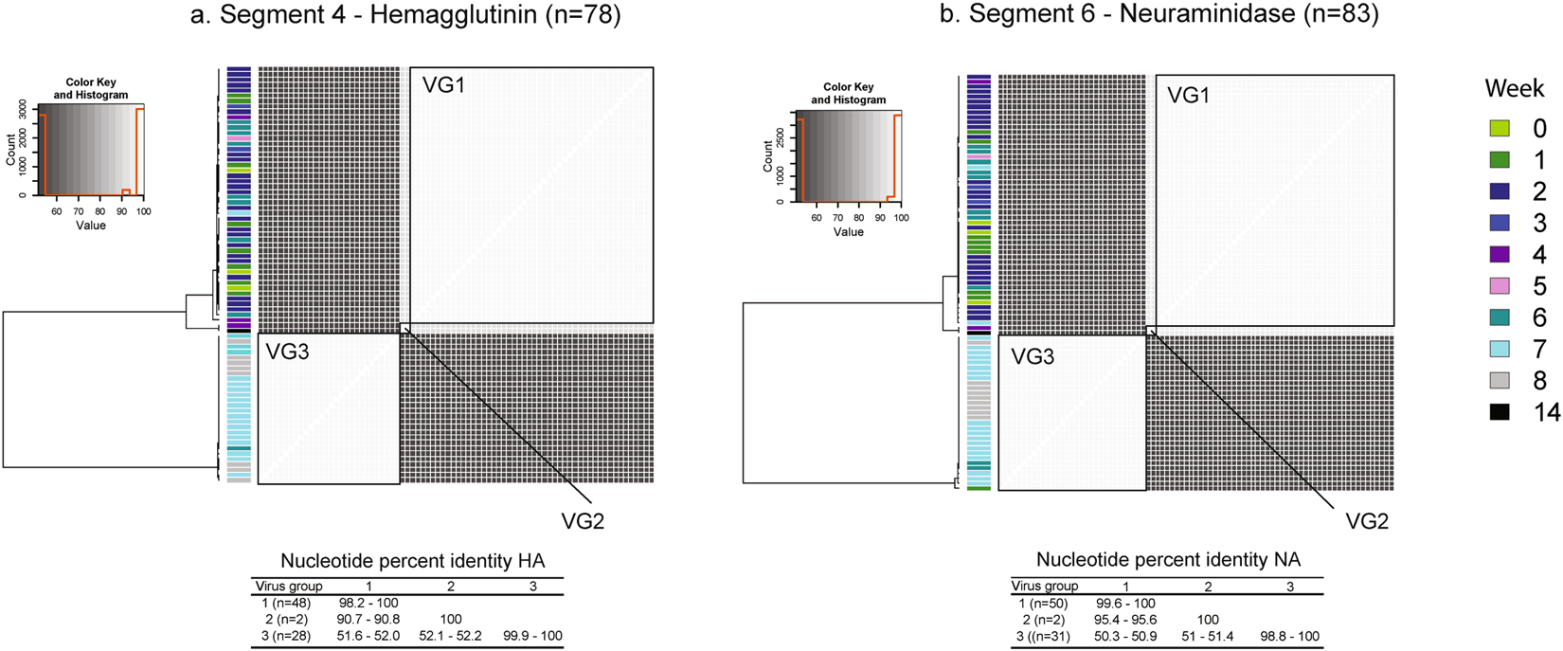
Hemagglutinin (HA) and neuraminidase (NA) pairwise sequence identity. Each heat map illustrates the percent sequence identity (ClustalW) among HA (**a**) and NA (**b**) sequences. The color key and histogram (x axis: percent identity; y axis: count) for each distance matrix is illustrated at the top left of each plot. Pairwise comparisons among virus group one (VG1, H1 gamma), two (VG2, H1 beta) and three (VG3, H3 cluster IV) are highlighted within black boxes. Dendrograms are distance based and do not illustrate the phylogenetic relationships between sequences. The bar side color at the left of each heat map indicates the sampling week (week 0 (W0) to week 14 (W14)) for each sample. The HA and NA pairwise comparison within and between groups is shown at the bottom of each plot.

**Figure 6:**
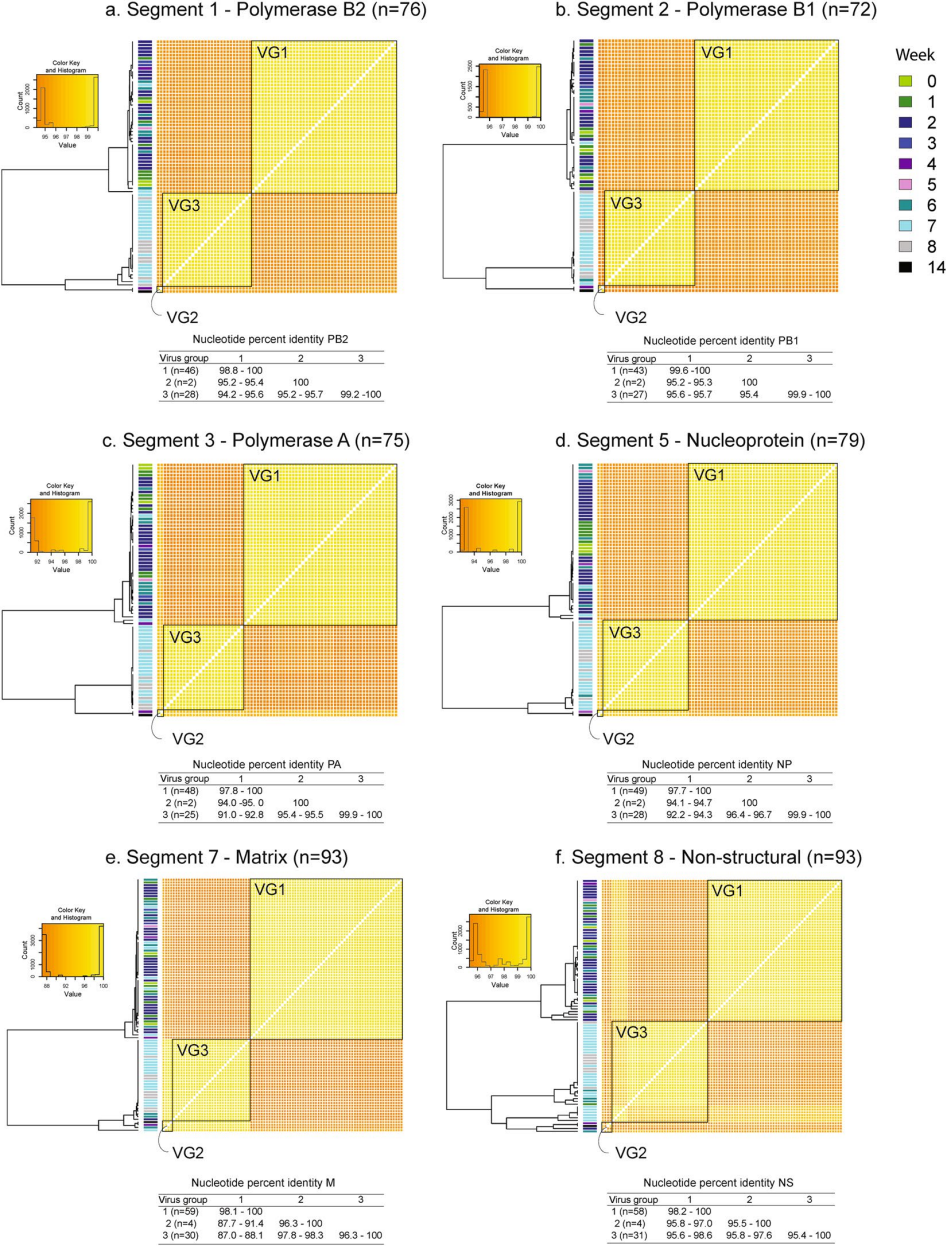
Pairwise sequence identity (ClustalW) among influenza A virus (IAV) internal genes. The color key and histogram (x axis: percent identity; y axis: count) for each distance matrix is illustrated at the top left of each plot. Pairwise comparisons among virus group one (VG1), two (VG2), and three (VG3) are highlighted within black boxes. Dendrograms are distance based and do not illustrate the phylogenetic relationships between sequences. The bar side color at the left of each heat map indicates the sampling week for each sample (week 0 (W0) to week 14 (W14)). The pairwise comparison within and between virus groups is shown at the bottom of each plot.

Out of all complete IAV gene segments obtained (n = 649), 78 (12%) and 83 (12.7%) were HA and NA sequences, respectively. The HA and NA pairwise sequence identity is illustrated in Fig. 5. HA VG1 (subtype H1 gamma) sequences (n = 48) were obtained between W0 and W7 and their percent sequence identity ranged between 98.2 and 100%. In contrast, HA VG2 (subtype H1 beta) sequences (n = 2) were only obtained at W4 and W14 and were 100% identical. Additionally, HA VG3 (subtype H3 cluster-IV) sequences (n = 28) were obtained only between W6 and W8 and their percent sequence identity ranged between 99.9% and 100%. Moreover, NA sequences (n = 83) included 50 sequences from VG1 (subtype N1), two from VG2 (subtype N1) and 31 from VG3 (subtype N2). NA sequences from VG1 were found between W0 and W7 while NA sequences from VG3 were assembled only at W1, W6, W7 and W8. The pairwise percent identity among NA sequences ranged between 99.6% and 100% for VG1 and between 98.8% and 100% for VG3. In contrast, NA sequences from VG2 (n = 2) were only assembled from W4 and W14 and were 100% identical. All sequences of each internal gene segment (genes 1,2,3,5,7 and 8) were aligned using ClustalW and also showed a clear distinction between VG1, VG2, and VG3 (Fig. 6).

### Diversity of hemagglutinin and neuraminidase proteins

**Figure 7:**
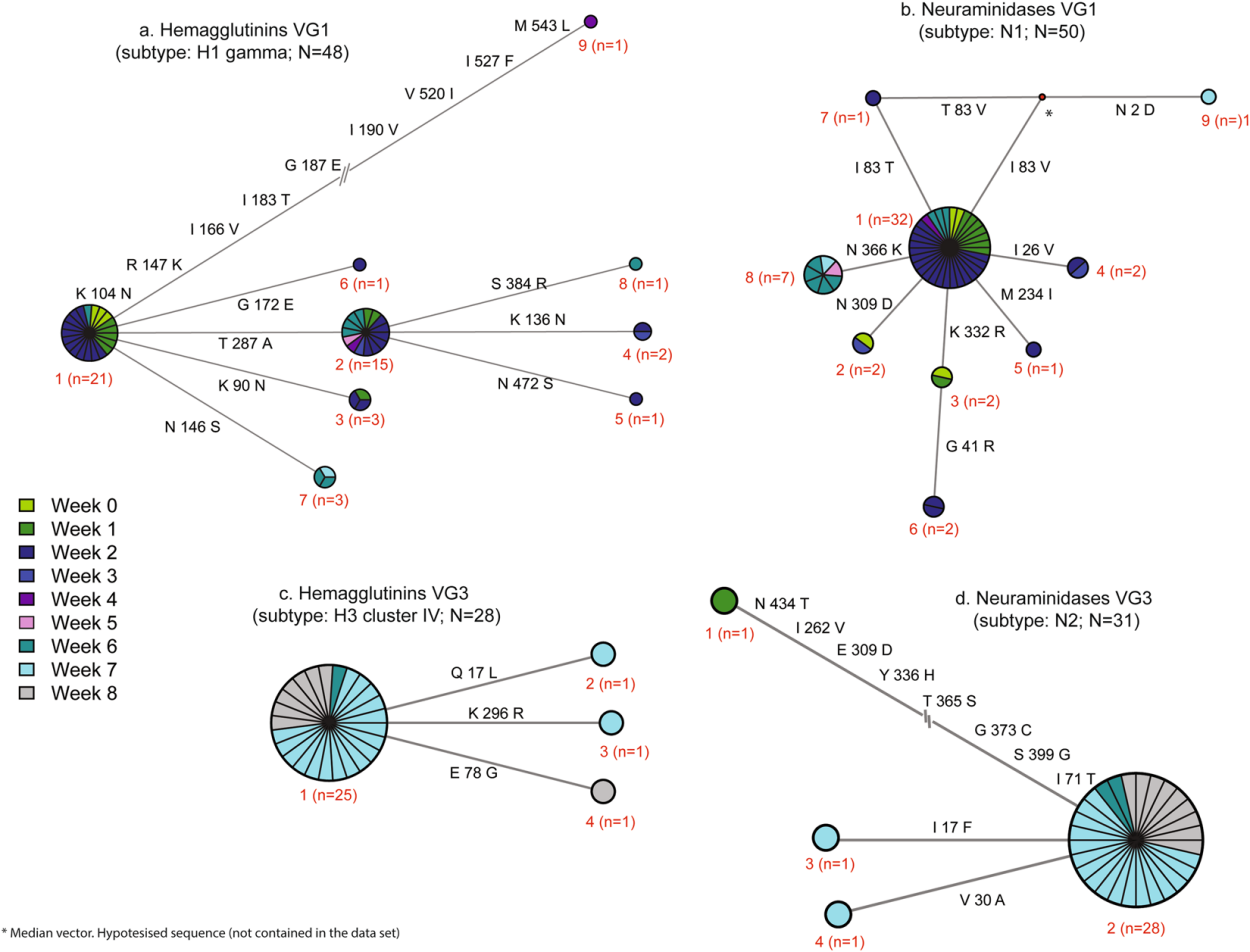
Network analysis of hemagglutinin (HA) and neuraminidase (NA) protein sequences of virus group one (VG1, H1 gamma) and three (VG3, H3 cluster IV). Network plots represent the relationships among HA and NA protein sequences for VG1 (panels a and b) and VG3 (panels c and d). Each node (circles) represents an amino acid sequence variant and its size is proportional to the frequency per node. Red numbers indicate the node number and sequence frequency (n). Nodes are color coded to indicate the week were each sequence variant was found. The distance between nodes is proportional to the number of amino acid differences between sequences (larger distances are indicated as “-//-“to fit the plot) and polymorphic sites between nodes are indicated (position and amino acid variant).

The hypothetical HA and NA proteins for VG1 and VG3 were translated to deduce the polymorphic amino acid sites within viral groups (Fig. 7). For VG2, the two HA and NA sequences identified at week four and 10 were 100% identical; thus, no further protein sequence analysis was performed. At W0 we only identified one VG1 HA variant (Fig. 7a, node 1). However, there were eight additional VG1 HA variants recovered during the study period (Fig. 7a, nodes 2 to 9). The majority of VG1 HA sequences (n = 36) corresponded to two specific amino acid variants (T287A) that are illustrated in Fig. 7a as node 1 (n = 21) and 2 (n = 15). The additional HA variants from VG1 differed in one to nine amino acids and were not found for more than 2 consecutive weeks. Additionally, nine VG1 NA variants were also identified. However, only two VG1 NA variants were recovered from more than two consecutive weeks (Fig. 7b, node 1 and 8).

In addition, four VG3 HA variants were identified (Fig. 7c) during the study period. The first and most common VG3 HA variant was first recovered at W6 and persisted until W8 (Fig. 7c node 1). The other three VG3 HA variants were only identified once during W7 and W8 (Fig. 7c, nodes 2 and 3). Moreover, the first NA protein from VG3 was identified at W1 and was not recovered again throughout the study period (Fig. 7d, node 1). The most frequent VG3 NA variant was first identified at W6 and proceeded for two additional weeks while the remaining two variants were found only at W7 (Fig. 7d).

### Recurrent detection of influenza A virus in pigs after weaning

**Figure 8:**
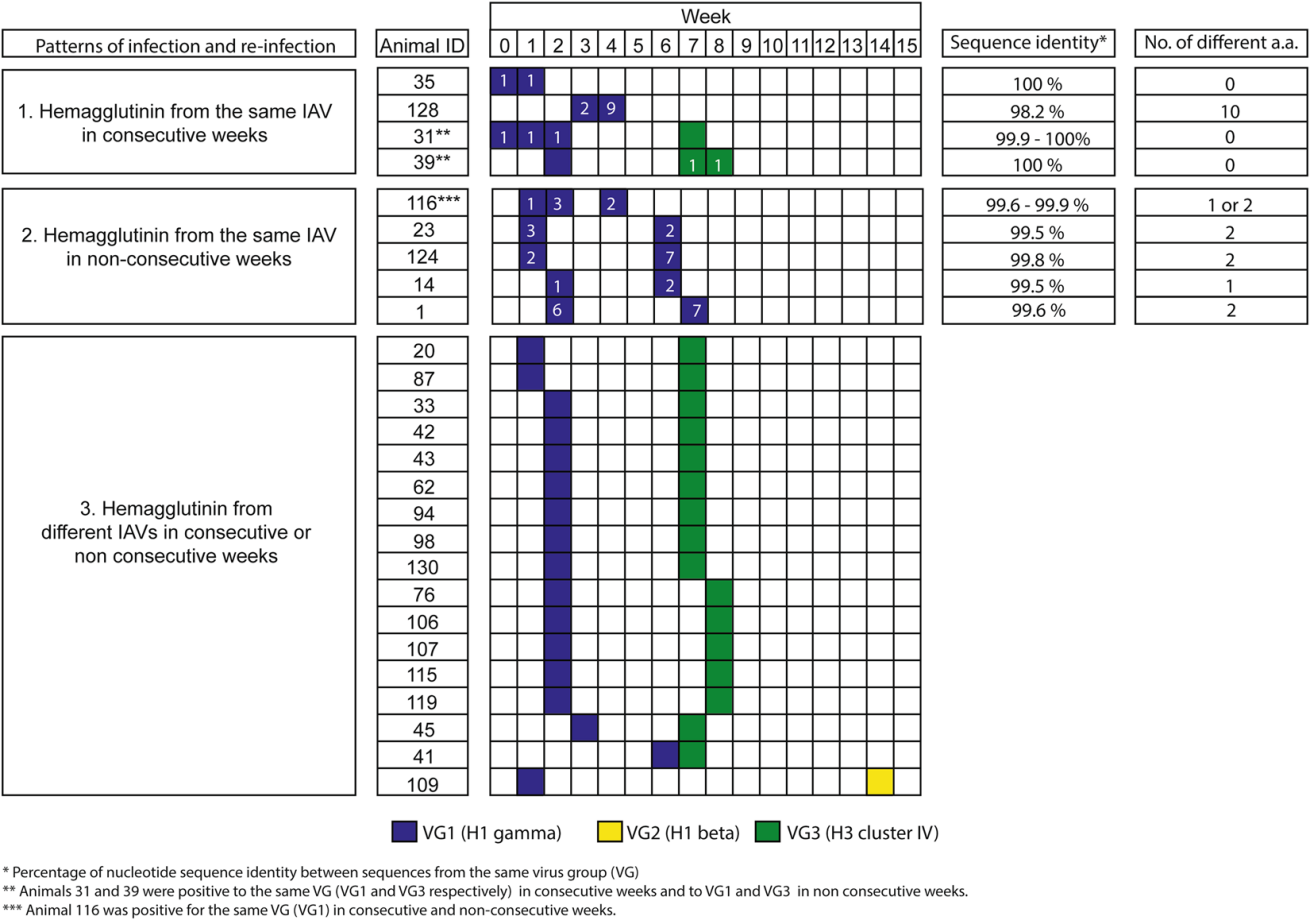
Patterns of influenza A virus (IAV) occurrence and recurrence. Fifty-six hemagglutinin (HA) nucleotide and amino acid sequences obtained from 26 pigs over time (week 0 to week 15) are illustrated based on virus group one (VG1, H1 gamma, blue), two (VG2, H1 beta, yellow), and three (VG3, H3 cluster-IV, green). Three different patterns of IAVs are shown: 1) HA sequences from the same IAV virus group found within a pig in consecutive weeks; 2) HA sequences from the same IAV virus group found within a pig in non-consecutive weeks; and 3) HA sequences from different IAV virus groups found within a pig in consecutive or non-consecutive weeks. White numbers within colored boxes indicate the HA node in which this sequence clustered at the amino acid level as indicated in Fig. 7.

To understand IAV occurrence and recurrence in pigs after weaning we compared the nucleotide and amino acid HA sequences of those samples sequenced from the same pig over time. For the purpose of this study, we defined occurrence as the detection of IAVs in a pig by RT-PCR in one or more consecutive weeks and recurrence as the detection of IAV in two or more non-consecutive weeks. Fifty-six samples collected from 26 pigs were used for this analysis (Fig. 8). Five out of these 26 pigs (19.2%) had IAVs from the same VG (VG1 or VG3) in consecutive weeks and the HA pairwise nucleotide sequence identity within pig ranged between 98.2% and 100%. Additionally, 23 out of these 26 pigs (88.5%) tested PCR positive twice in non-consecutive weeks meeting our definition of recurrent infection.

Complete HA sequences representing VG1 and VG3 were assembled from pig 41 at W6 and W7 respectively, and therefore animal 41 had also a recurrent IAV infection (Fig. 8). Most IAV recurrence (19 out of 24, 79.2%) happened with IAVs from different VGs (VG1 and VG3, n = 18; and VG1 and VG2, n = 1). However, five pigs (20.8%) had recurrent infections with VG1 IAVs. In these recurrent cases the percent of sequence identity within pig ranged between 99.6 and 99.9% and HA proteins between sampling events had at least one amino acid difference (Fig. 8).

## Discussion

To understand the persistence of IAVs in pigs we designed a prospective cohort study and followed 132 3-week old weaned pigs for 15 weeks. Using intensive sampling events and deep genome sequencing we were able to characterize with high resolution the epidemiological patterns of IAV infection, and the genetic and antigenic diversity of the viral population during infection of pigs. Strikingly we found the occurrence of two contiguous epidemic events of infection within a short period of time in which three different viral populations (viral groups) coexisted as a dynamic “swarm” of IAV gene alleles over time. Based on the study design and definitions, we demonstrated that pigs could have recurrent infections with IAVs that were closely related to each other or IAVs that were clearly distinct. Our findings unraveled a layer of IAV epidemiology and diversity not described previously. Understanding the genetic diversity and dynamics of IAV infections in pigs is crucial to design better health interventions to prevent and control the disease in pigs and to minimize the risk of transmission to people.

To our knowledge, this is the first prospective cohort study that evaluated the complete genome of IAVs during infection of a large population of pigs under field conditions. RNA viruses have higher mutation rates compared to many other microorganisms in nature^20,21^ and finding a “swarm” of IAV alleles closely related to each other at the population level was expected. The co-circulation of different IAV sequence variants during infection of people^13,22^, horses^23^, pigs^12,24^, and dogs^25^ has been demonstrated before. Additionally, the plasticity of swine IAVs at the individual level has been documented^12,24^ and supports our findings at the population level. However, we demonstrated that this “swarm” of IAV alleles in pigs could include both, closely related and clearly distinct genetic variants and subtypes. Furthermore, we found that this “swarm” of viruses could change dynamically over time and that the different gene segments could be detected at different proportions during epidemic events. The dynamic diversity of IAV genome over time may play a central role in the appearance of continuous infection and re-infection and should be further investigated to determine the mechanisms that allow one viral population to take over during epidemics.

In this study each epidemic event of infection was dominated by a single VG. Since pigs originated from a single farrow-to-wean source farm we speculate that all viruses originated from the same farm and finding positive pigs at arrival proved that the farrow-to-wean farm was a source of IAVs. However, we cannot rule out lateral introductions of IAVs to the cohort studied from other sources. While VG1 dominated the first epidemic event VG3 dominated the second one. However, we documented that certain gene segments of VG3 were detected earlier than others, and before than complete genomes could be recovered. Furthermore, we found a third VG (VG2) co-circulating with VG1 and VG3 without dominating any of the epidemic events. Surprisingly the complete genome of VG2 was only recovered when the prevalence of IAV infection was low (<4%) at W4 and W10. Interestingly VG2 belongs to the swine H1-beta viruses. The circulation of H1-beta viruses in swine has decreased significantly since the introduction of human IAVs into US swine populations in 2003. Overall, these findings indicate that some IAV alleles, might replicate hidden underneath a dominant cloud of IAV alleles, and can only be recovered when the epidemic event of dominant IAVs have ended and when high throughput genome sequencing technologies are used. We speculate that differential viral fitness of IAV alleles could be determined by virus competition from other co-circulating viruses or gene segments. A recent study in North America illustrated how new genetic lineages of swine IAVs have emerged without being noticed for several years^17^ while others that were commonly found before (e.g. H1 beta IAVs) now circulate unnoticed^9,17^. Therefore IAV fitness and even allele viral fitness of swine IAVs should be further investigated because they could determine the dynamics, emergence, and persistence of IAVs in swine populations.

IAVs are considered endemic in swine populations given their commonality and co-circulation of different subtypes. In the Midwestern USA, 90% of pig-production herds (with growing pigs) tested positive^16^ and in Europe IAVs are widely distributed in pig farms^26^. However, we speculate that the diversity and evolution of swine IAVs might be greatly underestimated or biased towards those genotypes that are over-represented during IAV epidemics or those that are selected during virus isolation^27^. Our approach to evaluate the complete genome of IAVs directly from the nasal swab samples using NGS technologies provided a greater resolution to characterize IAVs circulating in pigs. This approach could help to understand the mechanisms that allow IAVs to persist at the population level.

The segmented genome of IAV allows two or more viruses to exchange gene segments during infection (genetic reassortment)^1,28^ which is an important mechanism of virus diversification and emergence of novel IAV with zoonotic^29,30^ and pandemic potential^8^. In pigs, the antigenic genes are swapped between IAVs at different rates^31^ which indicates that IAV reassortment in pigs is not a random event. However, it is unknown whether reassortment is more likely to happen in certain stages of the IAV epidemics. Based on our results, it is possible that if a single VG dominates an epidemic, reassortment might be less likely to happen. However, if two epidemics overlap or co-infections happen when prevalence is low reassortment might be more likely because the proportion of each parental strain could determine reassortment rates. In other species, the frequency of genetic reassortment among IAVs closely related to each other is random^32^ while viruses evolving from very distinct genetic lineages are more restrictive^33^. Hence the reassortment rate of IAVs based on the proportion of different VGs should be further investigated. Virus diversification by reassortment has changed the genetic makeup of swine IAVs during the past three decades^10,11^ and allowed the emergence of the first human IAV pandemic of the 21^st^ century^34,35^.

Most pigs at weaning have some level of maternally derived antibodies (MDA) to different IAVs because IAVs are commonly found in farrow-to-wean herds. Hence, it is expected that the HA and NA will be under higher immune pressure after weaning compared to other viral gene segments given that HA and NA are the main antigenic proteins of the virus. In our study, the highest genetic diversity within IAV gene segments from the same VG was found for gene segment 7 (matrix) and 8 (non-structural) and not for segments 4 (HA) and 6 (NA). Furthermore, the distance matrix for the percent pairwise sequence identity of all IAV gene segments allowed us to differentiate the same three VGs identified using HA sequences. One previous study showed that all IAV gene segments, and not only HA and NA, present a dynamic distribution during infection of pigs with immunity to different IAVs^12^. Another study, demonstrated that the substitution rate for HA1 was not different between pigs with or without active immunity to IAVs^24^. Although, immune selection could depend on the affinity of antibodies to a given virus, IAV antigenic selection due to existing immunity is poorly understood and should be further investigated, especially to understand IAV immune evasion and re-infection in pigs after weaning.

For the purposes of this study we defined recurrent infection when IAV was detected twice in non-consecutive weeks given that duration of influenza infection in pigs lasts a maximum of 5 to 7 days^11^. Since only nasal swabs were collected and IAV isolation was not attempted, we cannot rule out the possibility of physical deposition of environmental IAVs rather than infection or re-infection. However, the epidemic events identified indicated that most pigs were indeed infected at least twice. Additionally, most cases of IAV recurrence happened with IAVs containing different antigenic subtypes (H1N1 and H3N2) suggesting that pigs were indeed re-infected, which is expected given the antigenic differences between H1 and H3 IAVs. Nevertheless, under our definition of recurrent infection we demonstrated that IAV recurrence could happen with viruses that differ at the HA level in only one or two amino acids. Whether these amino acid differences changed or not the antigenic properties of the virus and were responsible for IAV re-infection needs to be further investigated. One single amino acid difference within HA can change the antigenicity and receptor binding avidity of IAVs^36–38^. IAV re-infections have been described and characterized mostly in humans^39–41^. In pigs, the mechanisms that allow IAVs to re-infect an animal are not clearly understood. Multiple factors have been proposed which include host, environment and viral factors^42^ such as antigenic drift^38^, differences in cross protection among IAV phenotypes^43,44^, immune response^39,45^, MDA^46,47^ and the competition between naïve and memory B cells^48^.

This study also provides information on epidemiological findings relevant to what happens during IAV outbreaks in large swine populations. The number of prevalent cases in each epidemic peak at week 2 (W2) and 7 (W7) was not statistically different suggesting that the threshold point at which swine IAV epidemics subside was similar. In our study the transmission pattern for two distinct IAVs (VG1 and VG3) followed the same trend. This information is valuable because if transmission patterns of distinct IAVs are predictable then perhaps there could be similar interventions to prevent them.

We acknowledge the limitations of our study design. Our results might only represent the population of pigs from which the cohort was selected. However, the pig farm selected for this study represented a common commercial wean-to-finish farm in the USA and the epidemiological findings of this study are similar to prior studies in the USA^14,16^, Europe^49^, and field reports by veterinarians in the USA. Since there was limited information from the farrow-to-wean herd prior to the start of the study, we cannot rule out that VG2 or VG3 originated from the farrow-to-wean ﻿herd﻿. Overall, we found VG1 IAVs at the arrival of pigs and proved that pigs at weaning can be the source of IAVs to wean-to-finish farms. Additionally, our random sample selection and large sample size minimized the random error and allowed us to make conclusions at the population level. Moreover, the detection of IAV by RRT-PCR might not represent true infections, as it cannot differentiate the stage of IAV infection or whether replicating virus was present, which needs to be taken into consideration for all data interpretation. Additionally, it is possible that the estimated genetic diversity of IAVs during this study is biased because we only selected a set of positive samples and for certain weeks the proportion of samples sequenced might appear low. However, we sequenced 25% of all positive samples and performed the genome amplification directly from nasal swabs, which avoided genotype selection during virus isolation. The number of samples tested and sequenced in this study is probably the largest in any cohort study done to date in pigs. Finally, prospective cohort studies in pigs could also serve as an excellent animal model to study IAV transmission and evolution in humans, or to test the efficacy of health interventions given the physiological and immunological similarities between humans and pigs^50^, the homology between human and swine IAV subtypes, and the similarity on IAV transmission routes among humans and pigs^3,17,51^.

In conclusion, we demonstrated the complexity of IAV infections in pigs after weaning under field conditions. We illustrated the dynamic diversity of the IAV genome during infection of pigs and characterized the hypothetical antigenic proteins of the virus HA and NA. We showed that the prolonged persistence of IAVs in pigs could be the result of multiple IAV epidemic events that take place repeatedly over time or the re-infection with IAVs that are closely related to each other. These findings are important to control IAVs in pigs and to better understand virus diversity and emergence of IAVs in endemically infected swine populations.

## Methods

Protocols and procedures followed throughout the study were approved by the University of Minnesota Institutional Animal Care and Use Committee (IACUC 1207B17281), and the Institutional Biosafety Committee (IBC 1208H18341). The University of Minnesota IACUC adheres to the Animal Welfare Act as Amended (7 USC 2131-2156) regulation administrated by the United States Department of Agriculture (USDA).

A prospective pig cohort study was designed in a wean-to-finish site that receives pigs from a single farrow-to-wean farm. The source farm had history of endemic IAV infection but there was limited information on what viruses may have been in the herd prior to the beginning of the study. In the wean-to-finish site, pigs were raised in eight different barns from weaning (3 weeks of age) to market (24 weeks of age). Barns were filled within a week in three different arrivals and were managed all in/all out. 132 piglets were randomly selected at the first arrival of pigs to one of the barns. A total of 2200 piglets filled the barn and the selected cohort of pigs was maintained comingled with the other pigs in the barn (as distributed by the pig farmer after arrival). Sample size was estimated to be 95% confident to detect at least one IAV positive pig if the prevalence of infection was 2.5% or higher. Pigs were selected randomly by assigning a random number to all pigs in the first arrival and choosing those 132 pigs with the lowest random number assigned.

Each pig in the cohort was individually identified with an ear tag and individual nasal swabs (BBL CultureSwab, Becton Dickinson and Company, USA) were collected every week for 15 weeks. Swabs were refrigerated and transported to the laboratory within 6 hours of collection on the manufacture’s swab transport media. Within 24 hours of collection, swabs were placed into 1.8 ml micro centrifuge tubes with sample storing media (Dulbecco’s Modified Eagle Medium (DMEM), 5% antibiotic-antimycotic (Gibco, Life Technologies, USA containing 10000 IU/ml of penicillin, 10000 μg/ml of streptomycin, and 25 μg /ml of Fungizone), and 2% bovine serum albumin (BSA) fraction V 7.5% solution (Gibco, Life technologies, USA). Once in storage media, swabs were vortexed for 10 seconds and stored at −80 °C until IAV testing.

The nasal swabs were tested individually for swine IAVs by reverse transcriptase real time polymerase chain reaction (RRT-PCR) using protocols described elsewhere^52,53^. A sample was considered positive for IAV if the RRT-PCR cycle threshold (Ct) value was ≤35. The weekly prevalence rate (number of positive cases per week among pigs tested) and the period prevalence (number of pigs testing positive at least once during the 15-week study period) were estimated. The number of prevalent cases was compared between weeks and considered significantly different if the McNemars test p value was lower than 0.05. Additionally, the weekly incidence density (number of newly positive pigs at risk during a week) was estimated. Additionally, we estimated the number of weeks that the same pig tested positive for IAV and for the purposes of this study, we defined “recurrent infection” when IAV was detected in a pig two or more times during non-consecutive weeks.

A set of 92 RT-PCR positive swabs was selected for PCR amplification and deep genome sequencing. Swab sample selection targeted pigs with 2 or more positive swabs during the study period and the samples with the lowest Ct value because we expected to find in these samples a higher content of viral RNA. At least one positive sample per week was included. IAV genome was amplified directly from the nasal swabs in a single reaction using methods previously described^54^. Briefly, viral RNA was extracted from positive swabs using MagMax Viral RNA isolation kit (Ambion, Life Technologies, USA). One step RRT-PCR was performed using SuperScript III One-Step RT-PCR System with Platinum Taq DNA Polymerase (Invitrogen, Life Technologies, USA). A 100 μl PCR mix was prepared containing 20 μl DNase/RNase-Free distilled water (Gibco, USA), 50 μl 2x reaction mix, 2 μl SuperScript III RT mix, 2 μl (10 μM) of each primer (MBtuni12(M): ACGCGTGATCAGCRAAAGCAGG and MBtuni13: ACGCGTGATCAGTAGAAACAAGG), and 24 μl of RNA template. Gel electrophoresis was used to verify visually PCR amplicons. PCR products were purified using QIAquick Spin Kit (QIAGEN, USA), and eluted in 20 μl of DNase/RNase-free distilled water (Gibco, Life Technologies, USA). Samples were then submitted to the University of Minnesota Genomics Center (UMGC) for library preparation (TruSeq DNA HT sample prep kit, Illumina, USA) and sequenced using next generation sequencing (NGS) technologies (MiSeq paired end 250 cycles, Illumina, USA).

Sequencing quality control was first verified with FastQC^55^. Then Trimmomatic^56^ was used to trim low quality reads using the pair-end mode of the software. Sequencing assembly was performed using Bowtie2^57^ and SAMTools^58^ on a reference template containing 6 IAV internal gene references and 4 antigenic gene references (PB2 (CY099076.1), PB1 (CY099309.1), PA (CY045233.1), NP (CY009919.1), M (DQ150436.1), and NS (CY050162.1), H1 (FJ789832.1), H3 (KC992248.1), N1 (GU236519.1), N2 (KC866483.1)). If more than one IAV genotype was found, then different genome templates, obtained from the samples sequenced, were used to re-map the reads of all samples. The proportion of Illumina reads that were mapped to each IAV template was estimated by gene segment and week. Consensus sequences were established using the most common base at each nucleotide position (lower number of ambiguities) for each IAV gene segment and then trimmed to coding regions. Sequence functionality was verified using the NCBI FLu Annotation web-service (FLAN)^59^. Complete functional sequences from this final assembly were used to estimate IAV diversity during the study period. Furthermore, complete genomes were called when the eight complete gene segments were assembled. Mixed IAV infections were defined as samples where two or more complete consensus sequences for the same gene segment were obtained after mapping the Illumina reads to multiple IAV gene templates.

The main antigenic gene segments (HA and NA) were analyzed first and the antigenic subtype determined. Then, HA sequences were used to classify IAV into different viral groups (VG) based on the phylogenetic origins of H1 (gamma 1, gamma 2, delta 1, delta 2, alpha, or beta)^17^ or H3 (clusters I to IV)^10^ subtypes using the web-based tools available at the Influenza Research Database^60^. The number of virus variants for each VG was determined, and the persistence of the same VG at the population level was estimated over time. Then, all other gene segments were classified based on the HA designated VGs, and the genome constellation for each sample sequenced was inferred. Additionally, each set of IAV gene sequences (gene segments 1 to 8) was aligned using ClustalW^61^ and the pairwise distance identity was used to compare the viral diversity across gene segments using heat maps. Sequence assembly and analysis was done using the resources available at the University of Minnesota Supercomputing Institute (MSI). Statistical analysis and heat maps were performed using tabular methods and ggplots 2.17.0 in R.

Hypothetical HA and NA proteins were translated and amino acid sequences compared using median-joining network analysis^62^. First, all protein sequences were aligned to the most frequent sequence found during the study period using DNA-Alignment (Fluxus Technology Ltd, Germany). Then, polymorphic sites among antigenic proteins were estimated, polymorphic amino acids per site inferred and median-joining networks constructed^62^. Protein networks were drawn and annotated using Network 4.613, Network Publisher (Fluxus Technology Ltd, Germany), and Adobe Illustrator CC 2014 18.1.1 (Adobe Systems Incorporated, USA).

Finally, the patterns of recurrent ﻿IAV detections were compared among those pigs of which we sequenced more than one positive sample during the study period. The percent sequence identity of HA at the nucleotide level was used to estimate virus divergence within pigs over time and the findings compared to the different HA amino acid sequences found during the network analysis for HA proteins.
